# Using pedigree tracking of the *ex situ* metacollection of *Amorphophallus titanum* (Araceae) to identify challenges to maintaining genetic diversity in the botanical community

**DOI:** 10.1093/aob/mcaf038

**Published:** 2025-04-03

**Authors:** O G Murrell, Z Diaz-Martin, K Havens, M Hughes, A Meyer, J Tutt, N Zerega, J B Fant

**Affiliations:** Negaunee Institute for Plant Conservation and Action, Chicago Botanic Garden, Glencoe, IL 60022, USA; Plant Biology and Conservation, Northwestern University, Evanston, IL 60208, USA; Department of Natural Sciences, Manchester Metropolitan University, Manchester, M15 6BX, UK; Chester Zoo, Chester, CH2 1EU, UK; Negaunee Institute for Plant Conservation and Action, Chicago Botanic Garden, Glencoe, IL 60022, USA; Biology Department, Spelman College, Atlanta, GA 30314, USA; Negaunee Institute for Plant Conservation and Action, Chicago Botanic Garden, Glencoe, IL 60022, USA; Plant Biology and Conservation, Northwestern University, Evanston, IL 60208, USA; Royal Botanic Garden Edinburgh, Edinburgh, EH3 5LZ, UK; Botanic Gardens Conservation International (U.S.) Boylston, MA 01505, USA; Negaunee Institute for Plant Conservation and Action, Chicago Botanic Garden, Glencoe, IL 60022, USA; Department of Biology, University of Central Florida, Orlando, FL 32816, USA; Negaunee Institute for Plant Conservation and Action, Chicago Botanic Garden, Glencoe, IL 60022, USA; Plant Biology and Conservation, Northwestern University, Evanston, IL 60208, USA; Negaunee Institute for Plant Conservation and Action, Chicago Botanic Garden, Glencoe, IL 60022, USA; Plant Biology and Conservation, Northwestern University, Evanston, IL 60208, USA

**Keywords:** *Amorphophallus titanum*, titan arum, *ex situ* conservation, pedigree analysis, genetic diversity, endangered species, exceptional species, living plant collections

## Abstract

**Background and Aims:**

Rapid global biodiversity loss emphasizes the need to employ strategies that conserve the long-term viability of *ex situ* plant collections. A pedigree-based management approach is an effective strategy to track genetic diversity of living collections; however, its implementation requires accession-level data for all individuals across all botanic garden collections. Here, we use historic accession records to construct a pedigree and test how effective current protocols have been in managing *ex situ* diversity.

**Methods:**

We selected the titan arum, *Amorphophallus titanum* (Araceae), for this study, as it is exceptional, endangered, and has been globally held in collections for nearly 150 years. A pedigree-based data management approach would benefit the *ex situ* conservation of many similar species. Using accession data from nearly 1200 individual plants from 111 institutions worldwide, we constructed a pedigree to track the history of this species in collections and evaluate how well genetic diversity has been maintained in the metacollection.

**Key Results:**

We found that data and records for the *ex situ* metacollection of *Amorphophallus titanum* are severely lacking and are not standardized within the botanical community. Using the available data, we found that the metacollection is derived from few founders, material is rarely exchanged between institutions on different continents, and nearly a quarter of known crosses are between related individuals.

**Conclusions:**

Our work highlights the need for consistent, detailed record-keeping for effective implementation of an informed pedigree-based management approach and long-term maintenance of collections of endangered plant species in botanic gardens.

## INTRODUCTION

An estimated one million plant and animal species are threatened with extinction (IPBES, 2019), including 45 % of all flowering plants (Bachman *et al.*, 2024). This unprecedented worldwide decline in biodiversity necessitates conservation efforts that prevent the loss of remaining diversity. *Ex situ* conservation collections, maintained through zoos, aquaria and botanic gardens, are increasingly important for conserving species before they reach extinction in the wild (Conde *et al.*, 2011; Pritchard *et al.*, 2012; Wood *et al.*, 2020; Smith *et al.*, 2023). Conservation organizations must therefore establish protocols to manage endangered species to meet goals of sustainability and species recovery (Toone and Wallace, 1994; Ballou *et al.*, 2010; Seaton *et al.*, 2010; Santymire *et al.*, 2014; Fant *et al.*, 2016). For many plant species, *ex situ* conservation is achieved through seed banking (Dalrymple and Abeli, 2019; White *et al.*, 2023), which is a means to maintain a high number of individuals, maximize diversity and minimize selection. ‘Exceptional’ plant species, which either do not produce seeds or produce seeds that cannot be preserved in seed banks (Pence *et al.*, 2022), must instead be conserved as living specimens in *ex situ* collections (Fant *et al.*, 2016; Wood *et al.*, 2020). Approximately one-third of known exceptional species are threatened, making their effective conservation in *ex situ* collections critical and fundamental to preventing species loss (Pence *et al.*, 2022).

Exceptional species are often held in the collections of multiple botanic gardens and other conservation institutions, which necessitates a collective approach to management. The maintenance of living plants across multiple institutions presents a number of genetic and demographic challenges (Ballou *et al.*, 2010; Fant *et al.*, 2016). The zoo and aquarium communities have addressed these challenges by using pedigree-based approaches which have been shown to successfully manage animal species *ex situ* (i.e. in zoos) (Santymire *et al.*, 2014; Moran *et al.*, 2021). One of the main goals of these approaches is to use pedigree information to maintain genetic diversity in the metacollection over time to maximize the stability of the *ex situ* population. This is achieved by ensuring that the genetic diversity contributed by the founders is maintained across generations and that inbreeding is limited (Ballou *et al.*, 2010; Willoughby *et al.*, 2015). In the zoo and aquarium communities, a founder is defined as an individual brought into a collection from a source population (often the wild) that has no known relationship with the individuals in the *ex situ* population except for its own descendants.

Plant reproductive biology can present challenges to meeting this goal. In the botanical community, new plant material is often sourced from seed from few maternal lines, and as a result the origin of the collection comprises a number of related individuals. In addition, plants can often be propagated using asexual reproduction (via corm or bulb division, leaf cuttings, etc.), which can allow the rapid multiplication of individuals. This increases the size of collections and helps buffer against stochastic events associated with small population sizes (Ballou *et al.*, 2010); however, this increased redundancy can come at the cost of over-representation of a few founders from which material was cloned (Maunder *et al.*, 2001; Boakes *et al.*, 2007). Thus, large metacollections are not necessarily genetically diverse. Pedigree approaches can mitigate these risks and increase the chances of achieving desired diversity outcomes (Foster *et al.*, 2022).

The pedigree-based approach is slowly being adopted for the management of threatened plant species in botanic gardens (Wood *et al.*, 2020; Foster *et al.*, 2022; Diaz-Martin *et al.*, 2023). One of the barriers is that the accession- or individual-level data need to be collected across all the institutions that are holding specimens, representing the complete metacollection of the species (Griffith *et al.*, 2019). These pooled data can be used to create a pedigree, track accessions back to original founders, establish population sizes, and identify relatedness between individuals within and between collections. This information can then be used to make informed decisions to maintain genetic diversity and minimize inbreeding across the metacollection over time. To maintain captive populations, the zoological and aquarium communities manage individual-level data and share that information across institutions (e.g. Rabier *et al.*, 2020) using centralized databases [Species360 Zoological Information Management System (ZIMS)]. These databases offer a starting point for pedigree management, tracking lineages and founders over time and across populations (Species360, 2023). Further, these data can be transferred to pedigree management software (PMx) (Lacy *et al.*, 2012) to inform the breeding and exchange of individuals to maintain genetic diversity globally (Griffith *et al.*, 2020). Intentionally breeding unrelated and genetically under-represented lineages promotes metacollection-wide genetic diversity (Caballero and Toro, 2000; Boakes *et al.*, 2007). The use of a pedigree-based approach has led to the successful conservation and reintroduction of many animal species, such as the California condor and the black-footed ferret (Santymire *et al.*, 2014; Moran *et al.*, 2021). While proven to be a powerful approach to *ex situ* conservation, the pedigree approach is most effective when pedigrees are complete.

Identifying the risk of genetic diversity loss or inbreeding becomes more difficult when the metacollection houses hundreds or thousands of individuals across dozens of institutions, with each institution using its own approach to recording data. Such a metacollection can unknowingly have just a few founders and a high redundancy (i.e. clonality). Currently, examples of the use of the pedigree approach in the botanical community have focused on a handful of taxa that have had most of their individuals maintained by only a few institutions for only a few decades (Wood *et al.*, 2020; Foster *et al.*, 2022; Diaz-Martin *et al.*, 2023). In these cases, the risk of inbreeding and loss of genetic diversity can be easily tracked and characterized using institutional records. A larger number of institutions may produce greater differences in methods for collecting and maintaining accession data, complicating the implementation of pedigree approaches. This study evaluates our capacity to use a pedigree approach for an exceptional plant species held across numerous institutions and over a long period of time, which will provide insights into how the botanic garden community can improve *ex situ* management of endangered species in living collections going forward.

Here, we use titan arum (*Amorphophallus titanum*) to examine how well a pedigree approach can be applied to a large and historic metacollection. This species has been held in collections for 150 years and is maintained across numerous institutions worldwide. We posit two major research questions. First, can historic accession records be used to effectively manage *A. titanum* through a pedigree approach? Second, is the genetic diversity of the founders being maintained over time? To answer these questions, we constructed a pedigree using all available *A. titanum ex situ* accession data and tracked how frequently plants were cloned, crossed and exchanged between institutions. We provide important insights into how the botanic garden community can improve the management of the data necessary to conserve threatened exceptional plant species in *ex situ* metacollections.

## MATERIALS AND METHODS

### Study species

*Amorphophallus* (Araceae) consists of over 200 species ranging from Africa to South and Southeast Asia (Yudaputra *et al.*, 2022), but the most well-known among them is *Amorphophallus titanum*, an endangered species endemic to Sumatra, Indonesia (Yuzammi and Hadiah, 2018). There are an estimated 1000 individuals left in the wild; however, this number continues to decline, with one recent estimate suggesting there are now only 162 individuals remaining (Yudaputra *et al.*, 2022). *Amorphophallus titanum* faces many threats, including habitat fragmentation and loss, climate change and overharvesting of the corms (Yudaputra *et al.*, 2022). *Amorphophallus titanum* is exceptional and cannot be seed-banked, so living collections are the primary avenue for conservation. Flowering events are infrequent, short-lived and somewhat unpredictable, making crosses rare and difficult to coordinate. Additionally, *A. titanum* is protogynous, so pollen ripens only after the female phase of flowering has finished (Lobin *et al.*, 2007). Within-flower self-pollination is not thought to be possible for this species (Lobin *et al.*, 2007), but autogamy does occur in cultivation with human intervention and can sometimes result in viable seed, though fruit with no seeds may also form from such crosses. *Amorphophallus titanum* can also be propagated vegetatively by corm division, leaf cuttings or tissue culture (Lobin *et al.*, 2007). This charismatic plant is popular with the public and has been held in botanic garden collections for nearly 150 years (Lobin *et al.*, 2007). Today, we estimate that the species is held in ~250 conservation institutions worldwide. This is likely still an underestimate, as documentation is poor and there are numerous private collections.

### Pedigree data assembly

#### Accession data collection

To generate the pedigree of A. titanum ex situ, we requested any available accession data from 203 collections worldwide. We identified botanic gardens and universities using the Botanic Gardens Conservation International PlantSearch database of living collections [Botanic Gardens Conservation International (BGCI), 2021], the Global Biodiversity Information Facility (GBIF) database, Google searches (‘*Amorphophallus titanum*’, ‘botanic garden’, ‘titan arum’, etc.), and personal communication with researchers and botanic garden curators. Accession data were requested for any plants, living or dead, for which records existed (Supplementary Data Table S1).

#### Accession data assembly

We compiled all submitted accession data into a spreadsheet, with a separate record for each accession. If an accession included more than one individual plant, the entry was replicated with qualifiers so that each row represented only one individual. Therefore, each individual was assigned a database ID which served as the primary key to link all corresponding information (Supplementary Data Table S1). To test variation among continents, we assigned plants to one of three major continental collection groups: Asia and Oceania (AO), North America (AM), and Europe (EU). No data were received from South America or Africa. We received varied forms of raw data, including handwritten records, prose, lists and Microsoft Excel files. Institutional response rate was 55 %. Lack of existing data standards sometimes left us to interpret the information given, and we recognize this may have led to some misinterpretation. In the following sections we include the assumptions we made when imputing missing data.

### Identifying maternal and paternal lines

Pedigrees are useful for tracking individuals back to the founders of the population, but they require identifying the maternal (seed plant) and paternal (pollen donor) origins of each individual. Information on the sources and origins of individual plants is not often recorded and is easily lost over time. Even when seed sources are recorded, information about which individual’s pollen was used in a cross is often lacking. We tried to ensure that every parent plant was represented in our spreadsheet. In cases when data for those parental plants had not been submitted by the host institution, we pieced together parental lines using context clues, media coverage about flowering and pollination events, and references from other institutions. Some crosses of *A. titanum* individuals were well publicized by host gardens, with named parent plants that were known widely across the botanic garden community. In cases when the plant was recorded as having been collected from the wild, we simply designated the maternal and paternal plants as ‘WILD’.

### Pedigree analysis and the maintenance of genetic diversity

#### Number of founders

Founders create the baseline measure of genetic diversity in a metacollection, and the greater the number of genetically distinct founders, the greater the overall genetic diversity (Wood *et al.*, 2020). An important step in creating pedigrees is to track individual plants back to their original wild founders. We created a list of known expeditions that brought *A. titanum* from Sumatra into collections. There are doubtless more of these types of events than were recorded, given the poor documentation about them and the restrictions on collecting this species from the wild. We created a single source entry for each known expedition, as well as for all known commercial sources, thereby treating them as proxies for unique founding individuals. Researchers or collectors may have collected seeds from multiple plants, but detailed records about those expeditions were not available, so there was no way to verify. In most instances, we did not have enough information to determine how many plants were sampled from the wild or how they were distributed once they were collected. Considering the small wild population sizes of *A. titanum*, the rarity of flowering events, and the lack of information, we conservatively assumed that all plants (seeds or clones) from a given expedition were collected from only *one* plant, effectively identifying only one maternal line for each expedition event. While conservative, this assumption reduced the chances of overestimating diversity.

Following the protocol for wild-collected animals, we designated plants brought into botanic gardens from expeditions as having wild parents (Hogg *et al.*, 2019). The maternal line was labelled with the expedition name for tracking purposes. This designation assumed that (1) the maternal plant and the paternal plant were not related to each other; (2) parent plants from one expedition were not related to the parent plants from another expedition; and (3) all plants brought into a botanic garden from a given expedition were at least half-siblings. We acknowledge that given the small wild population sizes of *A. titanum*, the assumption that each seed was a half-sibling and therefore had different paternal parents, rather than assuming they were full-siblings and shared paternal parents, may have overestimated founder diversity (Hogg *et al.*, 2019).

**Fig. 1.**
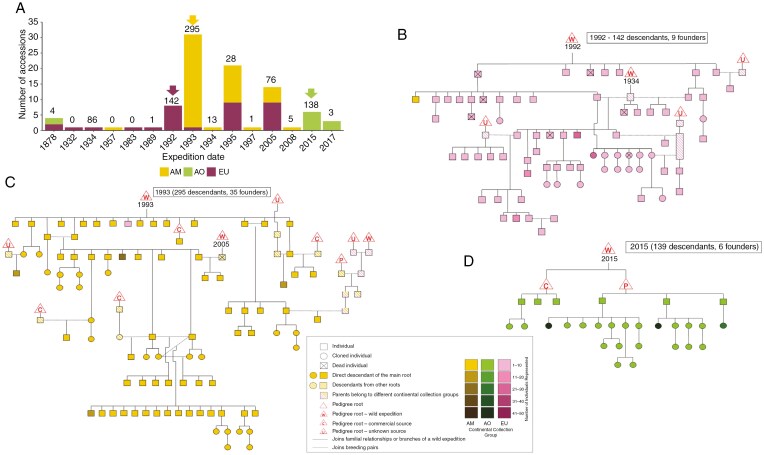
(A) Wild collection events from Sumatra and where the plants from these collection events were sent. Numbers on top of bars indicate the total number of offspring that can be traced directly back to the wild, while the *y*-axis indicates the number of new accessions brought into the *ex situ* metacollection from these expeditions. Arrows indicate the most prevalent wild collection events for each continental collection group. AM, North America; AO, Asia/Oceania; EU, Europe. (B–D) Largest pedigrees from each continental collection group of *A. titanum.* Colours indicate the number of individuals represented by each square or circle.

Whenever possible, we tracked all cultivated individuals back through the pedigree to expeditions and original commercial sources. When there was no provenance information, we listed the maternal and paternal accessions as ‘unknown’ and excluded those individuals from the pedigree. We were, and still are, unaware of a programme that could accommodate this type of dataset, particularly the inclusion of clones, so we assembled the accession data into a manually made pedigree using Microsoft PowerPoint (Fig. 1B–D).

### Reproduction within collections and exchanges between collections

We used the pedigree to evaluate how well crosses have carried the founding genetic diversity through generations, the prevalence of crosses, and how often crosses occurred between related individuals. If the data indicated that a plant originated from a corm split, vegetative propagation, leaf cutting or an ‘offshoot’, the plant was labelled as a clone. If the plant resulted directly from a cross of two individuals, it was categorized as non-clonal. If the plant was taken directly from the wild, it was also categorized as non-clonal (see the previous section). To estimate the degree of inbreeding, we used the accession data to count how many times related or potentially related individuals had been crossed.

**Fig. 2.**
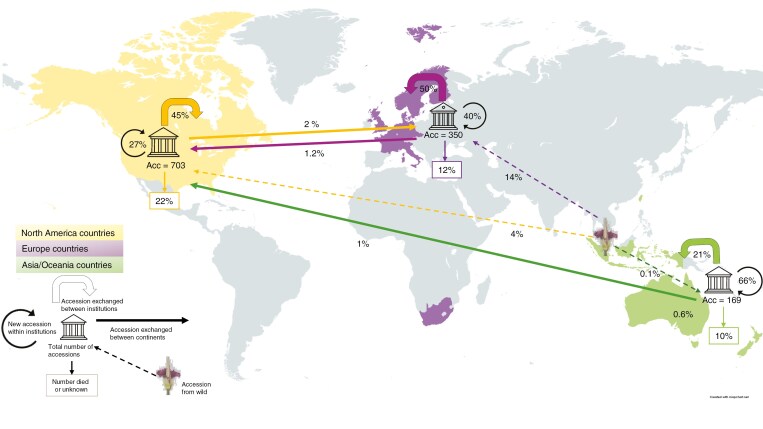
Distribution map showing the countries from which we received individual *ex situ* plant records. The arrows show the different ways that individuals were moved between continental collection groups. Acc, accessions.

To determine how often material had been propagated and exchanged between collections, we categorized the origin and propagule type of all plants into one of four categories: transfer (between institution), internal (within institution), wild origin, and unknown (Fig. 2) (Supplementary Data Table S1).

## RESULTS

### Pedigree assembly

We identified 250 conservation institutions and private collectors that potentially had *A. titanum* in their collections. We excluded the 24 private collectors due to the challenges of involving them in long-term conservation plans. We were able to contact 203 institutions. Of these, we received or pieced together data for 111 – an institutional response rate of 55 % (full list of institutions included can be found in Supplementary Data Table S2). Responding institutions provided accession data for 1188 individual plants, living and dead. We received the most accession records from North America (673), followed by Europe (348), with the fewest from Asia and Oceania (167) (Fig. 2). We did not receive responses from the few South American and African institutions that were listed on PlantSearch as having *A. titanum*. The only exception was one institution from South Africa, Stellenbosch University, whose plant originated from Botanischer Gärten der Universität Bonn and is therefore included in the European continental collection group for ease of analysis. Any gardens that are included in the dataset but did not have direct contact with us were included based on news articles or information from other gardens about the sources of their plants.

### Pedigree analysis and the maintenance of genetic diversity

#### Founders

We used wild expeditions in Sumatra as a proxy for the founders of the metacollection. We identified 18 wild germplasm collection events and therefore assumed we had 18 founding events. Data from 95 plants could be identified as resulting directly from these 18 events. Of these, 40 were either dead or of unknown status. The remaining 45 made up 0.9 % of the total individuals in collections. When we categorized the remaining plants by expedition (Fig. 1A), we found that most individuals (54 %) derived from two expeditions (1993 and 1995). With the exception of two expeditions (1878 and 1995), most plants derived from any given expedition remained within one continental collection group.

#### Reproduction within collections and exchanges between collections

To determine the contemporary inbreeding of *A. titanum* plants held *ex situ*, we evaluated clonality and the breeding between related individuals, using the pedigree. Of the 1188 individuals in our dataset, 287 were categorized as clones (24 %) of other individuals. We found evidence of 45 crosses, for which there were often too much missing data to determine the relatedness of the parents. Of the pairings we could track through the pedigree, there was a high prevalence of crosses between related individuals (27 %).

We also evaluated the status of *A. titanum* germplasm being maintained and transferred across institutions. Collections exchanged a range of material types (seeds, seedlings, whole plants, corms), with seeds being the most common propagation method. The highest incidence of missing data occurred when material was transferred. Additionally, of 579 documented transfers between institutions, 24 were intercontinental. Of the 45 documented crosses, none were between individuals from different continental collection groups. Slightly over half of the individuals were the result of crosses within the same institution. When inter-institutional crosses did occur, they were usually within the same continental region (Fig. 2). Given the rare intercontinental exchanges between collections, there may be limited overlap in genetic material.

## DISCUSSION

The long-term maintenance of genetic diversity in *ex situ* metacollections relies on informed management practices, such as a pedigree-based approach (Wood *et al.*, 2020). We used the available accession records of *A. titanum* held in the global metacollection to evaluate (1) the utility of historic accession records for creating a pedigree that supports an informed management approach, and (2) how well genetic diversity has been maintained for *A. titanum* over time in the *ex situ* metacollection. We attained available accession information from ~45 % of institutions holding the species, but it is important to note that the data we received were primarily from institutions in North America, Europe and Australia. There are numerous reasons for this bias, one of which is the factors that influence the global spread of botanic gardens, such as population size and GDP (Mounce *et al.*, 2017), but nested within this is a general lack of funding and infrastructure for smaller botanic gardens in much of the Southern Hemisphere.

We uncovered several gaps in the data that limited the utility of the pedigree management strategy. Overall, we found collection events were not well documented, and while many institutions could trace plants back to an original wild collection event, few had any data documenting how many maternal plants were sampled *in situ* and how those plants were distributed once collected. The paucity of detailed records limited both our ability to track genetic diversity and our accuracy in estimating the exact number of founders. The available data did, however, reveal moderate levels of clonality in a few genetic lineages. This suggests that genetic diversity is not evenly distributed in the metacollection and has led to high levels of redundancy. We also found a limited exchange of material between institutions, suggesting that there could be considerable divergence between continental institutions. In combination, these factors can lead to increased chances of inbreeding and potential loss of genetic diversity in the collection (Diaz-Martin *et al.*, 2023). Our work shows that assembling a pedigree for a species with a large, historic metacollection is challenging due to incomplete records. For this threatened species, our evidence suggests that moderate levels of clonality and few crosses between unrelated individuals have led to low diversity and elevated inbreeding across the metacollection, which is supported by a small molecular genetic study using double-digest restriction site-associated sequencing (ddRADseq; Peterson *et al.*, 2012) conducted on a subset of individuals (Murrell, 2023; an excerpt can be found in the Supplementary Data).

A major limiting factor for establishing a pedigree management approach for botanic gardens is the lack of standardized and consistent accession data collection across institutions. We found a lack of complete data throughout all stages of *ex situ* conservation of *A. titanum.* Records were often well organized within an institution but varied largely between institutions. Such inconsistencies limited our ability to estimate founders, track lineages and assemble an accurate pedigree. There are several botanic garden accession database systems (Iris, BGBASE, BRAHMS, Hortis, etc.) that use different data categories and varying definitions, exacerbating differences in how accession data are collected and managed. We strongly recommend that when plants are distributed data about the individuals be handled as carefully as the individual specimens themselves. Detailed, complete and standardized records should be made, properly stored, and distributed with the plants. One substantial roadblock we encountered was the lack of detailed information on wild sampling. Records are needed that include: the locations of individual plants sampled; the number of individuals in each population; which individuals were sampled (maternal lines); how material was sampled (seed, cutting, etc.); and where material was distributed after sampling. Without this information, the number of founders of an *ex situ* collection will not be certain. The updated PlantSearch pedigree module [Botanic Gardens Conservation International (BGCI), 2024] now accepts accession-level plant data, controlled by individual institutions. If widely used and kept up to date, this module can set the standard for recording accession data and facilitate its exchange. The use of molecular genetic approaches is needed to fill knowledge gaps and generate more accurate pedigrees. Given that genetic approaches are resource-intensive, not always possible, and difficult to scale, accurate accession records are essential data.

From the pedigree we created with available accession data, we evaluated how well botanic gardens have maintained genetic diversity in the *A. titanum* metacollection. We determined the prevalence of clones and the relative representation of founders in crosses. While clones retain the genetic diversity of founder genomes and create redundancy, certain lineages can be over-represented and inbreeding can increase (Moore *et al.*, 2008). Our pedigree identified that just under half of the accessions in collections were derived from asexual propagation. Given that most of these were from the same founder lineages, there is likely over-representation of very few lineages. Cloning is a valuable horticultural technique for increasing numbers and conserving genetic diversity, but without adequate record keeping it can increase the risk of inbreeding and dilute the contribution of a collection to diversity.

Using the pedigree, we estimated that fewer than one-third of crosses were between unrelated individuals. The rest were either selfed between inflorescences on the same plant (geitonogamy), between inflorescences of related individuals, or had insufficient data. While within-flower autogamy is uncommon for this species, it does occur in human-mediated crosses. Given these results, it is likely that the background level of inbreeding has increased over time (Diaz-Martin *et al.*, 2023), a conclusion supported by a small molecular genetic study conducted on these collections (Murrell, 2023). This molecular genetic study was conducted on only 65 plants and is therefore of limited utility, but it found that there was low genetic diversity and high inbreeding across all three continental collection groups and there were no significant differences between them (an excerpt can be found in Supplementary Data). A more extensive genetic study would be required to produce more significant evidence; however, when taken together, the findings of the pedigree and of the limited molecular genetic study highlight the need for a pedigree approach to effectively manage the *A. titanum* metacollection. This is challenging in part due to the scarcity of flowering events that limit opportunities for optimal crosses; however, it has been demonstrated that pollen can successfully be frozen and used for cross-pollination events (Wolkis *et al.*, 2024; J. Foster, Chicago Botanic Garden, USA, pers. comm.). The establishment of a pollen bank could facilitate exchange between institutions and across continents.

Foundationally, there is a need for standardized methods of collecting and recording accession data at all stages of *ex situ* conservation. The PlantSearch Pedigree module can help achieve this goal if it is widely used and continually updated. Global Conservation Consortia, coordinated by BGCI, are also in the process of serving as taxonomic champions for eight critically important taxa (*Acer*, cycads, dipterocarps, *Magnolia*, *Nothofagus*, oak, *Rhododendron* and *Erica*) (Pirie *et al.*, 2022); however, many exceptional species would benefit from this approach. Across zoos and aquaria, species-level data should be managed to ensure that all records of individuals across institutions are held in a centralized database that is accessible by request from other facilities (Wood *et al.*, 2020). We recommend that the botanic garden community adopt a similar approach to coordinating species management across institutions. Our work has made clear that previous passive management practices across the botanic garden community have negatively impacted the long-term sustainability of collections for one exceptional species, and we advocate the consideration of a pedigree approach to safeguard the conservation value of endangered species collections.

Finally, we urge conservation institutions to consider how their actions and horticultural practices may influence the genetic diversity of their collections. We offer five recommendations to help improve the status of the *ex situ* metacollection of *A. titanum* and other threatened species: (1) when sampling from the wild, the number of maternal founders and the ultimate destinations of these accessions are critical to document; (2) data must be standardized across botanic gardens and the PlantSearch Pedigree module can help facilitate this; (3) maternal and paternal plants must be tracked across institutions so that past cross information is known and related individuals are not crossed; (4) when individuals are transferred between institutions, in any form (seed or asexual propagation), accession-level data about the individual must accompany the germplasm to its new home; and perhaps most critically, (5) the botanic garden community needs to consider a consistent definition of ‘accession’, as we found that this term can refer to a single individual or a group of individuals brought into the collection at the same time. Through these recommendations, we hope to help the botanic garden community more effectively safeguard their threatened and endangered species well into the future.

## SUPPLEMENTARY DATA

Supplementary data are available at *Annals of Botany* online and consist of the following. Table S1: list of all data fields used to construct a pedigree, including data type, description, and two made-up example individuals and their associated data. Table S2: list of gardens and institutions with data included in this study, along with their country. Names are consistent with BGCI’s GardenSearch when possible. Genetic data supplement: methods and results of the small molecular genetic study, pulled from Murrell (2023).

mcaf038_suppl_Supplementary_Table_S1

mcaf038_suppl_Supplementary_Table_S2

mcaf038_suppl_Supplementary_Data
