# Supplementary material for: Using pedigree tracking of the ex situ metacollection of Amorphophallus titanum (Araceae) to identify challenges to maintaining genetic diversity in the botanical community
Source: Ann Bot. 2025 Apr 3;138(1):134–41. doi: 10.1093/aob/mcaf038 (PMC13409137; doi:10.1093/aob/mcaf038)
Supplement: mcaf038_suppl_Supplementary_Table_S1 [file mcaf038_suppl_supplementary_table_s1.docx]

| **Table S1** List of all data fields used to construct a pedigree, including data type, description, and two example individuals and their associated data. | | | | |
| --- | --- | --- | --- | --- |
| Column name | Data type | Description | Example record 1 | Example record 2 |
| Database_ID | Accession | Unique ID given to each individual for accession into this dataset. If an accession included more than one individual plant, the entry was replicated with qualifiers so that each row represented only one individual. | AT_EXBG_0001 | AT_SEBG_0002 |
| Institution | Accession | The institution that holds the plant that sent data and/or germplasm to us for inclusion in the analysis (home institution) | Example Botanic Garden | Second Example Botanic Garden |
| Country | Accession/Categorical | The country of the home institution | USA | England |
| CCG | Categorical | The continental collection group of the home institution | NorthAmerica | Europe |
| Genus | Accession | Genus of the accession – always “Amorphophallus” | Amorphophallus | Amorphophallus |
| Species | Accession | Species of the accession – always “titanum” | titanum | titanum |
| Source_ID | Accession | The accession number of the plant at the home institution | 67521*A | 2010-0002 |
| Accession_Date | Accession | The date the home institution accessioned the plant to their collection | UNK | 6/3/2010 |
| Exchange | Categorical | How the plant came into the current collection: **transfer** (inter-institutional exchange), **internal** (i.e. cross or clone), from **wild**, or **UNK** | Transfer | Internal |
| Exchange_Type | Categorical | Origin of the plant that was accessioned into the current collection (i.e., **asexual** = corm, cutting, etc.; **sexual** = seedling, seed, pollen, etc.; **UNK**; “plant” could refer to a sexual or asexual Exchange Type, depending on original propagation method) | UNK | Asexual |
| Source_Type | Categorical | A more detailed description of Exchange Type (i.e., asexual = **corm**, **cutting**, etc**.**; sexual = **seedling**, **seed**, **pollen**, etc.) | UNK | corm |
| Clonal_NonClonal | Categorical | Original origin of the plant [sexual = **non-clonal (NC)** always; however, asexual = NC when plant or corm is originally derived from a cross, or = **CLONE** when originally derived from a corm split or a tissue cutting where original plants still exist; **UNK**; **WILD**] | NC | CLONE |
| Source_Location | Accession | Institution where the accession came into the current collection | Botanic Garden | Second Example Botanic Garden |
| Origin | Accession | Whether the plant was cultivated (**C**), wild (**W**), the progeny of a wild individual or individuals (**Z**), or unknown (**UNK**) | Z | Z |
| Maternal_Accession | Accession | The mother plant’s **accession number** at its home institution; **WILD**; **UNK** | “Titan” | Split from 2003-5410 |
| Maternal_ID | Accession | The **Database_ID** that corresponds to the maternal plant; **WILD**; **UNK** | AT_BOTG_0425 | AT_SEBG_0002X |
| Cross_Location | Accession | Where the cross or propagation event took place that resulted in the current accession | Botanic Garden | Second Example Botanic Garden |
| Paternal_Accession | Accession | The father plant’s **accession number** from its home institution; if clonal, paternal accession is labeled “**CLONE**”; **WILD**; **UNK** | Mr. Arum | CLONE |
| Paternal_ID | Accession | The **Database_ID** that corresponds with the paternal plant; if clonal, paternal ID is “**CLONE**”, **WILD**, **UNK** | AT_BOTG_0677 | CLONE |
| Birth_Date | Accession | The date the plant germinated, was crossed, or was propagated. If this information is unknown, then this is listed as the accession date | 2001 | 6/13/2010 |
| Wild_Collector | Accession | If applicable, the name of the collector who collected or sampled this accession from the wild | NA | Jane Doe |
| Wild_Collection_Location | Accession | The location in Sumatra where the accession was collected.  If the accession is known wild origin but no specific location was given, “Sumatra, Indonesia” was used. | NA | Sumatra, Indonesia |
| Wild_Collection_Date | Accession | The date the wild collection event took place | NA | 1991 |
| Alive_Dead | Accession/Categorical | Whether the plant is alive or dead at its home institution | alive | alive |
