# Supplementary material for: Using pedigree tracking of the ex situ metacollection of Amorphophallus titanum (Araceae) to identify challenges to maintaining genetic diversity in the botanical community
Source: Ann Bot. 2025 Apr 3;138(1):134–41. doi: 10.1093/aob/mcaf038 (PMC13409137; doi:10.1093/aob/mcaf038)
Supplement: mcaf038_suppl_Supplementary_Table_S2 [file mcaf038_suppl_supplementary_table_s2.docx]

| **Table S2** List of gardens and institutions with data included in this study, along with their country. Names are consistent with BGCI’s GardenSearch when possible. | |
| --- | --- |
| Institution Name | Country |
| Ambler Arboretum of Temple University | United States |
| Arboretum and Botanical Garden at Cal State Fullerton | United States |
| Atlanta Botanical Garden | United States |
| Bergen Botanical Garden (Universitetshagene) | Norway |
| Bergius Botanic Garden | Sweden |
| Bogor Botanic Gardens (BRIN) | Indonesia |
| Botanical Garden Liberec | Czech Republic |
| Botanischer Gärten der Universität Bonn | Germany |
| Botanischer Gärten der Carl von Ossietzky-Universitat Oldenburg | Germany |
| Botanischer Gärten der Ruhr-Universität Bochum | Germany |
| Botanischer Gärten der Universität Basel | Switzerland |
| Botanischer Gärten der Universität Bern BOGA | Switzerland |
| Botanischer Gärten der Universität Kiel | Germany |
| Brooklyn Botanic Garden | United States |
| Cambridge University Botanic Garden | United Kingdom |
| Chester Zoo | United Kingdom |
| Chicago Botanic Garden | United States |
| Cleveland Metroparks Zoo | United States |
| College of Science and Health Greenhouse, William Patterson University | United States |
| Como Park Zoo and Conservatory | United States |
| Conservatoire et Jardin botaniques de la Ville de Genève | Switzerland |
| Cornell Botanic Gardens | United States |
| Daniel Stowe Botanical Garden | United States |
| Dartmouth Life Science Center Greenhouse | United States |
| Denver Botanic Gardens | United States |
| Die Flora der Botanische Garten Köln | Germany |
| Dr Cecelia Koo Botanic Conservation Center | Taiwan |
| Duke Biology Plant Teaching and Research Facility | United States |
| Dunedin Botanic Garden | New Zealand |
| E.W. Heier Teaching and Research Greenhouses, Binghamton University | United States |
| Eastern Illinois University Thut Greenhouse | United States |
| Eden Project | United Kingdom |
| Foster Botanical Gardens | United States |
| Frederik Meijer Gardens & Sculpture Park | United States |
| Ganna Walska Lotusland | United States |
| Garfield Park Conservatory | United States |
| Greater Des Moines Botanical Garden | United States |
| Greenhouses at Dr. David G. Carter Science Building, Eastern Connecticut State University | United States |
| Greenhouses at McCardell Bicentennial Hall, Middlebury College | United States |
| Harold L. Lyon Arboretum | United States |
| Herrenhäuser Gärten | Germany |
| Hortus Botanicus Amsterdam (Amsterdam Botanic Gardens) | Netherlands |
| Hortus Botanicus Leiden | Netherlands |
| Indiana University Biology Building Greenhouse | United States |
| Jardin Botanique de la Ville et de l'Université de Caen | France |
| Jardin Botanique de Montréal | Canada |
| Jardins botaniques du Grand Nancy et de l'Université de Lorraine | France |
| Juniper Level Botanic Garden | United States |
| Kansas State University Gardens | United States |
| Lauritzen Gardens | United States |
| Les Serres du Jardin Botanique | Belgium |
| Longwood Gardens | United States |
| Marie Selby Botanical Gardens (Selby Gardens) | United States |
| McMaster Biology Department Greenhouse | Canada |
| Mercer Botanic Gardens | United States |
| Miami University Belk Greenhouse | United States |
| Mississippi State University | United States |
| Missouri Botanical Garden | United States |
| Mitchell Park Domes | United States |
| Montgomery Botanical Center | United States |
| Moore Farms Botanical Garden | United States |
| Mount Lofty Botanic Garden | Australia |
| Myriad Botanical Gardens | United States |
| Naples Botanical Garden | United States |
| National Tropical Botanical Garden | United States |
| New York Botanical Garden | United States |
| Niagara Parks Botanical Gardens | Canada |
| Norfolk Botanical Garden | United States |
| Nussbaum Science Center, Taylor University | United States |
| Oak Park Conservatory | United States |
| Ökologisch-Botanischer Garten Bayreuth | Germany |
| Orange Coast College Horticulture Garden Lab | United States |
| Paignton Zoo | United States |
| Palmengarten der Stadt Frankfurt | Germany |
| Papiliorama Nocturama Tropical Gardens | Switzerland |
| Phipps Conservatory and Botanical Gardens | United States |
| Plantentiun Meise (Meise Botanic Garden) | Belgium |
| Plantentiun Universiteit Gent (Ghent University Botanical Garden) | Belgium |
| Rollins College Greenhouse | United States |
| Rotterdam Zoo and Botanical Garden (Diergaarde Blijdorp) | Netherlands |
| Royal Botanic Garden Edinburgh | United Kingdom |
| Royal Botanic Garden Sydney | Australia |
| Royal Botanic Gardens, Kew | United Kingdom |
| San Antonio Botanical Garden and Mitzi Weems | United States |
| San Diego Zoo Botanical Gardens | United States |
| San Francisco Conservatory of Flowers | United States |
| Science & Engineering Building, Washington State University | United States |
| Stellenbosch University Botanical Garden | South Africa |
| The Arboretum at Gustavus Adolphus College | United States |
| The Arnold Arboretum of Harvard University | United States |
| The Botanic Garden of Smith College | United States |
| The Greenhouse and Greenroof of St. Olaf College | United States |
| The Huntington Library, Art Museum and Botanical Gardens | United States |
| The Ohio State University Biological Sciences Greenhouse | United States |
| The Ruth Warren Abbott Greenhouse, Southwestern College | United States |
| The Spheres | United States |
| Toronto Zoo | Canada |
| Tucson Botanical Gardens | United States |
| UC Santa Barbara Biology Greenhouse | United States |
| United States Botanic Garden | United States |
| University of California Botanical Garden at Berkeley | United States |
| University of Connecticut Arboretum | United States |
| University of Helsinki Botanic Garden | Finland |
| University of Minnesota College of Biological Sciences Conservatory & Botanical Collection | United States |
| University of Missouri St. Louis Biology Greenhouse | United States |
| University of North Carolina Charlotte Botanical Gardens | United States |
| University of Rhode Island Botanical Gardens | United States |
| University of Washington Botanic Gardens | United States |
| University of Wisconsin-Madison Botany Garden and Greenhouse | United States |
| Waimea Valley Arboretum and Botanical Garden | United States |
| Western Illinois School of Agriculture Greenhouse | United States |
