# Supplementary material for: Using pedigree tracking of the ex situ metacollection of Amorphophallus titanum (Araceae) to identify challenges to maintaining genetic diversity in the botanical community
Source: Ann Bot. 2025 Apr 3;138(1):134–41. doi: 10.1093/aob/mcaf038 (PMC13409137; doi:10.1093/aob/mcaf038)
Supplement: mcaf038_suppl_Supplementary_Data [file mcaf038_suppl_supplementary_data.docx]

Extracted from Murrell OG. 2023. Adopting a Zoological Pedigree Approach to Botanic Garden Collections: The Status of the Worldwide Ex Situ Collection of *Amorphophallus titanum* (Araceae). Master’s Thesis. Northwestern University.

**Genetic Data Assembly**

A genetic analysis was conducted on a subset of individuals using double digest restriction site-associated sequencing (ddRADseq) in order to confirm the findings from the pedigree and to calculate indices of population genetic diversity. We collected silica-dried germplasm samples (mainly leaf tissue, but some corm or spathe tissue was also collected, depending on availability) from as many ex situ conservation collections as possible. To test the conclusions drawn from the pedigree we subsampled these collections. To choose the samples that would be included in the libraries, we randomly sampled 25 individuals from North America, 25 individuals from Europe, and 20 samples from Asia and Oceania using a random number generator. All individual samples that we received from Asia and Oceania were included due to the small sample size.

Genomic DNA was extracted from the silica-dried leaf samples using a modified DNeasy Plant Mini Kit (Qiagen, Venlo, Netherlands) protocol. DNA concentrations for each sample were quantified using a Qubit fluorometer with the dsDNA Broad-Range Assay Kit (Waltham, MA, USA). We then followed a modified double-digestion restriction site-associated sequencing (ddRADseq) protocol (Peterson *et al.*, 2012). The DNA concentrations from two individuals from Asia and Oceania were too low to use, reducing the sample size of Asia and Oceania to 18 individuals. We digested the genomic DNA of these samples using the restriction enzymes EcoRI and MspI (New England Biolabs, Ipswich, MA, USA). The full library was split into two halves. The first half of the samples were ligated with EcoRI cutsite-specific barcodes and a MspI adapter. The second half of the samples received the same unique barcodes but received a second MspI adapter so that the two distinct libraries could be distinguished. We used the BluePippin system (Sage Science, Beverly, MA, USA) to run 1.5% agarose gel electrophoresis that selected sequences between 250-600 bp (The Field Museum of Natural History, Chicago, USA). We then amplified both libraries through polymerase chain reaction (PCR). The DNA concentrations of each library were then quantified using the Qubit fluorometer with the dsDNA High-Sensitivity Assay Kit (Waltham, MA, USA). The final libraries were sent to the Northwestern University School of Medicine Sequencing Core, where they were checked for the correct size distribution using an Agilent Bioanalyzer (Santa Clara, CA, USA) and sequenced using pair-read 150-bp sequencing (Illumina NovaSeq 6000 platform).

***SNP calling and filtering***

STACKS v. 2.2 (Catchen *et al.*, 2011, 2013) was used to call single nucleotide polymorphisms (SNPs) *de novo.* First, the individuals were separated based on their unique barcodes, and the libraries were quality-filtered using the command *process_radtags*. For quality filtering, the command was set to remove barcodes with up to two mismatches (-adapter_mm), rescue barcodes with up to one mismatch (-r), remove any uncalled bases (-c), discard reads with low quality (-q), truncate final read length to 100 bases (-t), and set the score limit to 10 (-s). We then optimized the parameters -m, -M, and -n using the denovo_map.pl pipeline. Five individuals from each continental collection group were selected for use in this process. The different parameters did not greatly affect the outputs. Genetic distance in the MDS plots, the number of SNPs, and the level of genetic diversity were all comparable across parameters. Therefore, the default parameters were used (-m 2, -M 3, -n 2). The final SNP calling was accomplished through the same denovo_map.pl pipeline, but all samples were used to create the catalog. All individuals were assigned their proper continental collection group in the population map (--popmap). We also set the denovo_map.pl to require a minimum allele frequency of 0.05 (--min_maf) and to retain one SNP per stack to minimize linkage disequilibrium (--write-random-snp).

Lastly, we used VCFtools 0.1.16 (Danecek *et al.*, 2011) to quality filter SNPs. We filtered the dataset twice: once without considering Hardy-Weinberg equilibrium (HWE), and again considering HWE. Because both datasets produced nearly equivalent results, only the filtering methods that included a HWE filter are included here. Individuals with more than 90% missing data were excluded, along with loci that contained more than 70% missing data (--max-missing), and the minimum mean depth of coverage was set to 10X (--minDP). SNPs with a depth of coverage above 57.88 (two standard deviations above the mean DP) were also excluded (--max-meanDP). Sites across the entire genomic dataset with a p-value below a threshold of 0.05 were assumed to be out of HWE and were excluded (--hwe) (Catchen et al. 2011, 2013; Pearman et al. 2022).

**Genetic Analysis**

***Founders***

To further estimate the number of founders beyond the pedigree analysis, we utilized the molecular co-ancestry method of Nomura (2008) with NeEstimator v2 (Do *et al.*, 2014) to calculate the effective population size (Ne) for each continental collection group individually, and for the whole group of samples treated as one population. The range of effective population sizes across groups was used as an estimate of founders. To quantify the amount of genetic diversity in collections, we used R Statistical Software (v. 4.1.3; R Core Team, 2022) and the package *hierfstat* (Goudet, 2005) to calculate gene diversity (Hs). To determine if differences between any two continental collection groups were statistically significant, we performed three paired t-tests between each continental collection group. Individual-level heterozygosity (PHt) was calculated using the R function GENHET (Coulon, 2009), on which a Kruskal-Wallace test was performed, using a Bonferroni correction for the p-values. The number of private alleles (Pa) and total number of variant and polymorphic sites were calculated by the *populations* program in STACKS.

***Inbreeding in Collections***

To evaluate inbreeding in collections, we use the R package *hierfstat* (Goudet, 2005) to calculate inbreeding (FIS) per continental collection group. Individual-level inbreeding (F) was calculated using PLINK 2.0 (Purcell and Chang, 2023; Chang *et al.*, 2015) with the --het tag. A Kruskal-Wallace test was also performed (with Bonferroni correction) on the values of F to determine if F was significantly different between groups. We calculated FST using VCFtools using --weir-fst-pop.

***Uniqueness of continental collection groups***

To quantify the uniqueness of continental collection groups and to find the number of distinct genetic populations, we compared diversity measures across continental groups and used ADMIXTURE (Alexander *et al.*, 2009) to determine if the geographic populations match the genetic populations. The program ADMIXTURE was used to construct a maximum likelihood estimation of the number of populations and individual ancestries by considering varying levels of K from 1-10 and evaluating the best fit using a cross-validation procedure. The value of K with the lowest cross-validation score was treated as the best fit for the data. The divergence between populations was evaluated using a principal component analysis (PCA) in which the first two axes were kept using the R package *adegenet* (Jombart, 2008). The diversity and inbreeding measures calculated in the previous section were calculated for each continental collection group individually to determine whether any one group was more or less diverse or inbred than the others.

**Genetic Analysis Results**

***Datasets***

After the quality filtering was complete, we were left with 65 total individuals for genetic analysis: 23 from Europe, 25 from North America, and 17 from Asia and Oceania. 706 SNPs (1,412 alleles) were kept out of a possible 9,591. We performed analysis before and after filtering the SNPs for Hardy-Weinberg equilibrium (HWE). When quality filtering did not include HWE, 8,090 SNPs were kept. When filtering included HWE, 706 SNPs were kept. The dataset that was not filtered for HWE produced equivalent results and is not reported here. In keeping only loci that showed HWE, we made assumptions that all individuals comprise a complete population. Following the findings of Pearman *et al.* (2022), we acknowledge that the usage of an approach that removes genetic loci that depart from HWE across the entire dataset has likely reduced the amount of population structure. However, choosing to not filter for Hardy-Weinberg equilibrium is likely to lead to the retention of genotyping errors and loci that may be under selection (Pearman *et al.*, 2022).

***Founders***

Using the pedigree, we identified 18 wild expeditions (founders). We utilized the genetic data to calculate effective population size (using NeEstimator) which is an estimate of the number of individuals in the population that are contributing to the genetic diversity of that population. The effective population sizes of all three continental collection groups were smaller than the total sample size (Table S3). In general, lower Ne when compared to N indicates that fewer individuals are contributing to the genetic diversity of the population than there are individuals in the population. The confidence intervals vary widely, but it is known that when hundreds or thousands of SNPs are used, precision decreases in estimates of Ne (Do *et al.*, 2014). Ne can also be an imprecise measure itself but is useful for comparing values across populations.

The range of Ne can be used as an estimate for the number of individuals that contributed to the diversity of the current population. In an ideal pedigree, all founders would be contributing equally to the population. If all founders are contributing equally to the population, the number of founders and Ne will be equivalent, if not, Ne and number of founders will diverge. Given this, there are an estimated 2-20 founders (Table S3), given the range of Ne across all three continental collection groups. This is in the range of our estimate of 18 founders from the pedigree.

***Contemporary diversity and inbreeding in collections***

To evaluate the contemporary diversity of the collections and how well that diversity is being maintained, we calculated several genetic diversity measures using the 706 SNPs that were quality-filtered and filtered for HWE. To evaluate the genetic diversity in the global collection of *A. titanum*, we evaluated gene diversity (Hs) and individual-level heterozygosity (PHt), both for the metacollection (Table S3) and for each continental collection group (Figure S1).All values of PHt values were similar (no significant differences, P_AM-AO_ = 0.77; P_AM-EU_ = 1.0; P_AO-EU_ = 0.15) among continental groups, with Asia and Oceania having the highest value. Similarly, all values of Hs were not significantly different from one another ((P_AM-AO_ = 0.93; P_AM-EU_ = 0.99; P_AO-EU_ = 0.92). Asia and Oceania again showed the greatest value for gene diversity. This suggests variability in the Asia and Oceania continental collection group.

We quantified the maintenance of this genetic diversity by calculating inbreeding. We found values of inbreeding to be positive and high for the global population (Table S3) and for each continental collection group (Figure S2), both at the locus (FIS) and individual level (F), suggesting some level of inbreeding at all levels. There were no significant differences between values of F across continental collection groups (P_AM-AO_ = 0.78; P_AM-EU_ = 0.99; P_AO-EU_ = 0.14). There were significant differences between values of FIS across continental collection groups (P_AM-AO_ < 0.001; P_AM-EU_ = 0.003; P_AO-EU_ < 0.001); however, FIS was positive for both North America and Europe but was very slightly negative for Asia and Oceania (mean Fis = -0.02).

***Uniqueness of continental collection groups***

Using the program ADMIXTURE to identify structure within collections, we found that two ancestral populations (K = 2; CV = 0.67), and three ancestral populations (K = 3; CV= 0.67) were equally the best fit, so only K = 3 is reported here (Figure S3). We can conclude that there are likely three predominant ancestral sources of genetic material (Lawson *et al.*, 2018). All ancestral sources are present on each continent, but some individuals derived from a single ancestral source. For instance, Asia and Oceania had a genetic group not well represented in Europe or North America. Overall, the plot indicates that there is weak genetic structuring in collections. Without wild sampling, we cannot be sure if this reflects genetic structure of just collections or the wild populations broadly. ADMIXTURE is influenced by having related individuals in the dataset, so the groupings identified could represent maternal lines, expeditions, or wild population structure. There is not a way to distinguish between these with this dataset. The results of the principal components analysis (PCA) show little evidence of genetic structure (Figure S4), supporting the conclusions from ADMIXTURE. Additionally, calculations of pairwise FST (Table S4) from PMx are low and the estimated mean FST for the global population was 0.054, indicating small, insignificant differentiation between populations.

**Tables and Figures**

| **Table S3.** Genetic measures for the metacollection and each continental collection group. Values for N, Ne, F, and Hs were calculated both from the genetic data only and by PMx(**). PHt and FIS were calculated from genetic data alone.  *CI (95%) – Jackknife on samples  **calculated in PMx. For F and Hs, values reported in parentheses were calculated using PMx. The first value in the parentheses was calculated using only accession data. The second value in the parentheses was calculated using both accession data and genetic data (pairwise relatedness matrix). N, or total number of individuals in the population, in PMx only includes living individuals. | | | | |
| --- | --- | --- | --- | --- |
| **Genetic Measure** | **Total** | **AM** | **AO** | **EU** |
| N | 65 | 25 | 17 | 23 |
| Ne (founders) | 21.2  *20.9-21.6 | 12.6  *8.1-20.3 | 1.8  *1.0-3.9 | 18.7  *6.7-767.6 |
| N** (living only) | 1126 | 620 | 188 | 318 |
| Founders** | 29, 40 | 20 | 4 | 15 |
| Mean inbreeding (F) | 0.16  (0.17; 0.14) | 0.18  (0.21; 0.19) | 0.03  (0.00; 0.00) | 0.23  (0.05; 0.04) |
| Locus-level inbreeding (FIS) | 0.10 | 0.14 | -0.02 | 0.18 |
| Gene diversity (Hs) | 0.37  (0.89; 0.90) | 0.37  (0.81; 0.81) | 0.37  (0.25; 0.33) | 0.37  (0.81; 0.82) |
| Individual-level heterozygosity (PHt) | 0.32 | 0.31 | 0.37 | 0.30 |

***
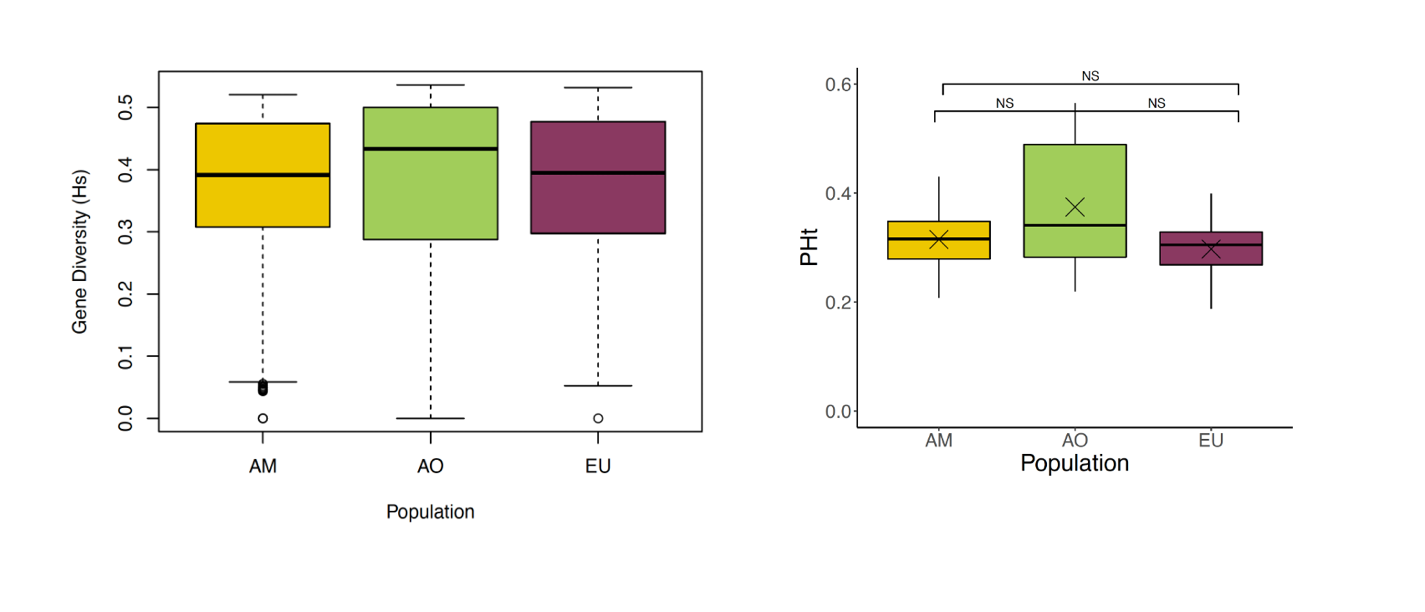
*Figure S1.** Gene diversity (Hs) (P_AM-AO_ = 0.93; P_AM-EU_ = 0.99; P_AO-EU_ = 0.92) and individual-level heterozygosity (PHt) (P_AM-AO_ = 0.77; P_AM-EU_ = 1.0; P_AO-EU_ = 0.15) calculated for each continental collection group using 706 quality filtered SNPs from 65 individuals.

*
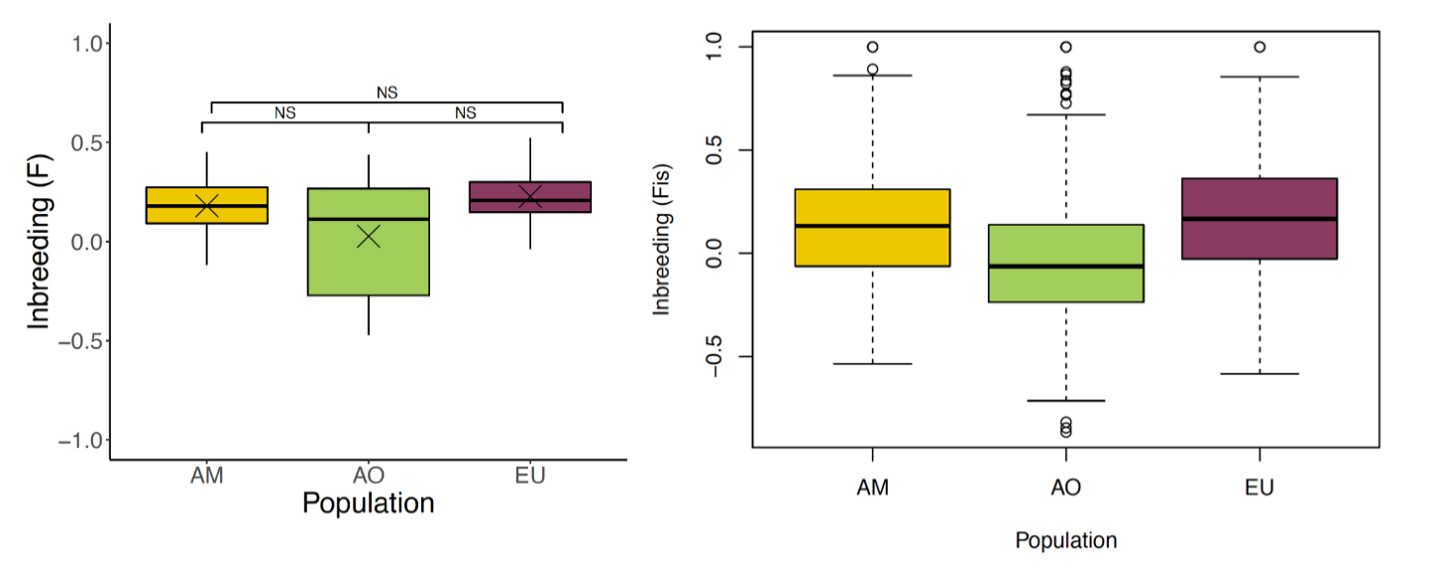
*

**Figure S2.** Inbreeding at both the individual (F) ( P_AM-AO_ = 0.78; P_AM-EU_ = 0.99; P_AO-EU_ = 0.14) and locus level (FIS) ( P_AM-AO_ < 0.001; P_AM-EU_ = 0.003; P_AO-EU_ < 0.001) for each continental collection group, calculated using 706 quality filtered SNPs from 65 individuals.

**
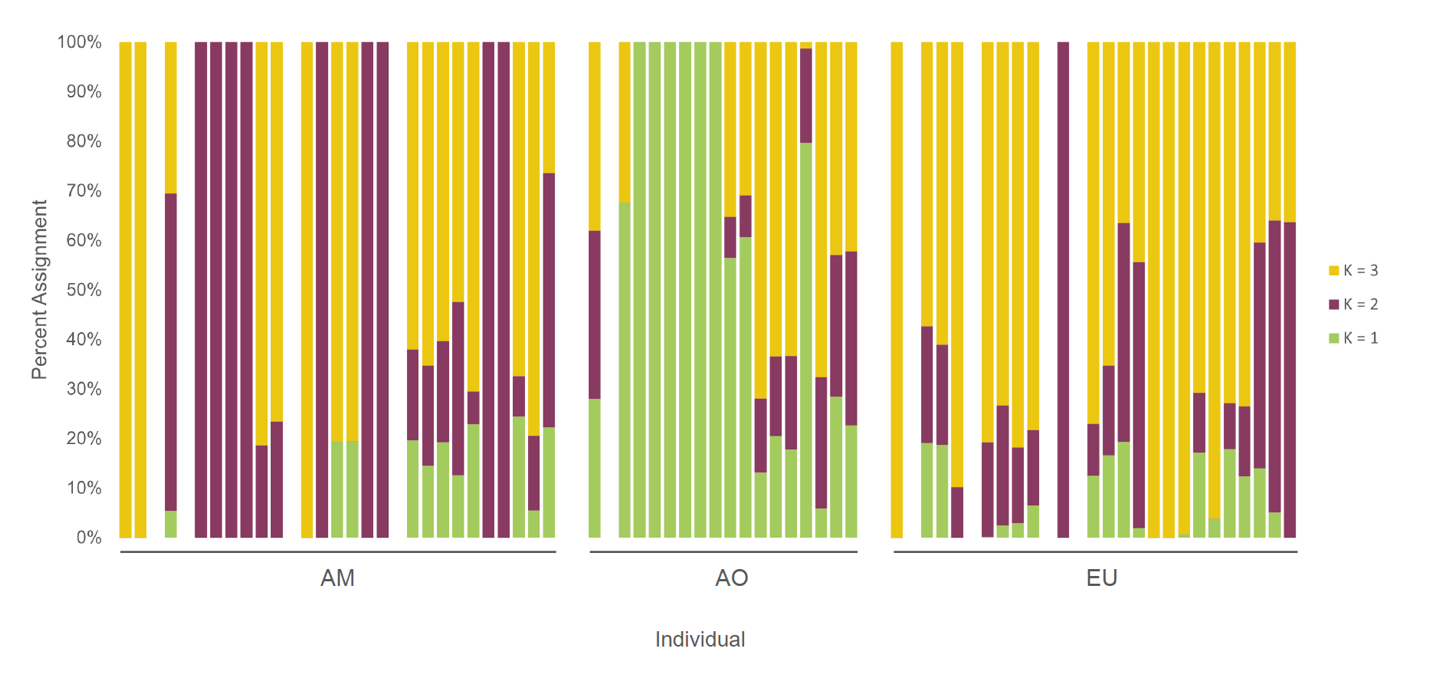
**

**Figure S3.** ADMIXTURE plot for K = 3. Each column represents an individual, and they are grouped by continental collection group as indicated by the bars below the plots (AM, AO, EU), and grouped by source (as far back as we could track). Each source is separated by a space. This plot was generated from 706 quality-filtered SNPs genotypes from 65 individuals.

| **Table S4.** Pairwise FST between each continental collection group calculated with PMx | | |
| --- | --- | --- |
|  | **EU** | **AM** |
| **AM** | 0.10 |  |
| **AO** | 0.13 | 0.06 |

| **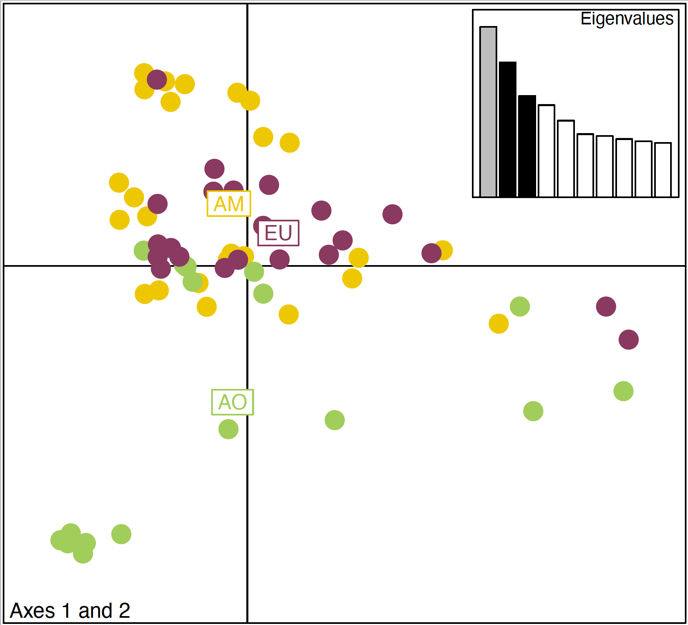**  **Figure S4.** Axes 1 and 2 of the principal components analysis (PCA) and accompanying eigenvalues conducted on the dataset of 706 quality filtered SNPs from 65 individuals. |
| --- |

**References**

Alexander DH, Novembre J, Lange K. 2009. Fast model-based estimation of ancestry in

unrelated individuals. *Genome Research* 19: 1655–1664.

Catchen JM, Amores A, Hohenlohe P, Cresko W, Postlethwait JH. 2011. Stacks: building

and genotyping Loci de novo from short-read sequences. *G3 (Bethesda, Md.)* 1: 171–182.

Catchen J, Hohenlohe PA, Bassham S, Amores A, Cresko WA. 2013. Stacks: an analysis tool

set for population genomics. *Molecular Ecology* 22: 3124–3140.

Chang CC, Chow CC, Tellier LC, Vattikuti S, Purcell SM, Lee JJ. 2015. Second-generation

PLINK: rising to the challenge of larger and richer datasets. *GigaScience* 4: 7.

Coulon A. 2010. genhet: an easy-to-use R function to estimate individual heterozygosity.

*Molecular Ecology Resources* 10: 167–169.

Danecek P, Auton A, Abecasis G, *et al.* 2011. The variant call format and VCFtools. *Bioinformatics (Oxford, England)* 27: 2156–2158.

Do C, Waples RS, Peel D, Macbeth GM, Tillett BJ, Ovenden JR. 2014. NeEstimator v2: re-implementation of software for the estimation of contemporary effective population size (Ne ) from genetic data. *Molecular Ecology Resources* 14: 209–214.

Goudet J. 2005. hierfstat, a package for r to compute and test hierarchical F-statistics. *Molecular Ecology Notes* 5: 184–186.

Jombart T. 2008. adegenet: a R package for the multivariate analysis of genetic markers.

*Bioinformatics (Oxford, England)* 24: 1403–1405.

Lawson DJ, van Dorp L, Falush D. 2018. A tutorial on how not to over-interpret

STRUCTURE and ADMIXTURE bar plots. *Nature Communications* 9: 3258.

Nomura T. 2008. Estimation of effective number of breeders from molecular coancestry of

single cohort sample. *Evolutionary Applications* 1: 462–474.

Pearman WS, Urban L, Alexander A. 2022. Commonly used Hardy–Weinberg equilibrium

filtering schemes impact population structure inferences using RADseq data. *Molecular Ecology Resources* 22: 2599–2613.

Peterson BK, Weber JN, Kay EH, Fisher HS, Hoekstra HE. 2012. Double digest RADseq: an

inexpensive method for de novo SNP discovery and genotyping in model and non-model species. *PloS One* 7: e37135.

Purcell S, Chang C. 2023. PLINK 2.0. Available from www.cog-genomics.org/plink/2.0/

R Core Team. 2022. R: A language and environment for statistical computing. R Foundation for

Statistical Computing, Vienna, Austria. Available from https://www.R-project.org/
